# Transposition element MERVL regulates DNA demethylation through TET3 in oxidative-damaged mouse preimplantation embryos

**DOI:** 10.1186/s10020-025-01143-3

**Published:** 2025-03-12

**Authors:** Lihong Liu, Siyao Ha, Dan Cao, MingQing Li, Zhiling Li

**Affiliations:** 1 Reproductive Center, The First Affiliated Hospital of Shantou University Medical College, Shantou University, Shantou, 515041 China; 2 Institute Obsterics and Gynecology, Hospital of Obsterics and Gynecology,Fudan University, Shanghai, 200080 China

**Keywords:** MERVL, Oxidative damage, DNA methylation, TET3

## Abstract

Transposable elements (TEs) comprise approximately half of eukaryotic genomes and significantly contribute to genome plasticity. In this study, we focused on a specific TE, MERVL, which exhibits particular expression during the 2-cell stage and commonly serves as an indicator of embryonic totipotency. However, its precise role in embryo development remains mysterious. We utilized DRUG-seq to investigate the effects of oxidative damage on genes and TEs expression. Our findings revealed that exposure to hydrogen peroxide (H_2_O_2_) could induce DNA damage, apoptosis, and incomplete DNA demethylation in embryos, which were potentially associated with MERVL expression. To further explore its function, antisense nucleotides (ASO) targeting MERVL were constructed to knockdown the expression in early embryos. Notably, this knockdown led to the occurrence of DNA damage and apoptosis as early as the 2-cell stage, consequently reducing the number of embryos that could progress to the blastocyst stage. Moreover, we discovered that MERVL exerted an influence on the reprogramming of embryonic DNA methylation. In MERVL-deficient embryos, the activity of the DNA demethylase ten-eleven translocation 3 (TET3) was suppressed, resulting in impaired demethylation when compared to normal development. This impairment might underpin the mechanism that impacts embryonic development. Collectively, our study not only verified the crucial role of MERVL in embryonic development but also probed its regulatory function in DNA methylation reprogramming, thereby laying a solid foundation for further investigations into MERVL's role.

**Supplementary Information:**

The online version contains supplementary material available at 10.1186/s10020-025-01143-3.

## Introduction

Does assisted reproductive technology (ART) increase the long-term health risk of offspring? What is the mechanism? For a long time, this question remained a complex puzzle. ART has emerged as a beacon of hope for approximately 8 million infertile families (Crawford and Ledger 2019), yet concerns regarding the well-being of the offspring it generates persist. The in vitro culture milieu, encompassing factors such as culture medium composition, temperature, light exposure, and oxygen concentration, fails to completely simulate the intricate intrauterine developmental environment. This discrepancy potentially triggers an overproduction of reactive oxygen species (ROS), culminating in oxidative damage (Scherrer et al. 2015; Fleming et al. 2018). Notably, compared to naturally conceived progeny, ART offspring exhibit elevated risks of cardiovascular and metabolic disorders (Guo et al. 2017; Cui et al. 2020), an increased susceptibility to childhood cancer and neurodevelopmental impairments (Kallen et al. 2010; Lo et al. 2022). A prominent theory posits that ART has the propensity to perturb epigenetic modifications, particularly DNA methylation, both in the fetus and placenta (Barberet et al. 2022). ART-related epigenetic mechanisms target gene networks pivotal for embryonic development, neurodevelopment, growth, and metabolic pathways (Cannarella et al. 2022). Regrettably, deciphering the molecular underpinnings by which adverse early embryonic factors translate into long-term health risks remains an arduous task.

Clinical investigations have shown that antioxidant levels rise at four distinct stages during ART procedures, namely before and after egg extraction and embryo transfer. This indicates that patients experience varying degrees of oxidative stress (Ozatik et al. 2013). Our research team have been exploring the effects of oxidative stress on in vitro fertilization (IVF)-derived embryos. To simulate the oxidative damage of zygotes in clinical IVF treatment, we treated mouse fertilized eggs with different concentrations of hydrogen (H_2_O_2_) and found that 0.03 mM H_2_O_2_ was the lowest effective concentration, reducing the proportion of blastocyst formation by inducing DNA damage and aneuploidy. This finding closely resembles the clinical phenomenon of embryo oxidative damage in IVF (Qian et al. 2016; Huang et al. 2019). This stage is significant for gene transcription and genome-wide epigenetic reprogramming. During this period, environmental variations and external influences may lead to the manifestation of epigenetic inheritance.

During mammalian embryonic development, the genome undergoes two momentous phases of DNA methylation pattern reprogramming, namely post-fertilization and following germline cell specification (Greenberg and Bourc’his 2019; Singh et al. 2023). The zygotic genome activation (ZGA), a remarkable epigenetic remodeling process, occurs and involves global DNA demethylation, chromatin remodeling, genome spatial reorganization, as well as substantial transcriptional changes (Eckersley-Maslin et al. 2018). DNA methylation plays an essential role in various biological processes, in ensuring chromosomal stability, facilitating genomic imprinting, and mediating X-chromosome inactivation via transcriptional regulation (Yamazaki et al. 2020). DNA methyltransferase (DNMT) and TET enzymes are responsible for establishing and erasing DNA methylation (Wu and Zhang 2014). After fertilization, in male pronucleus, the ten-eleven translocation 3 (TET3) enzyme oxidizes 5-methylcytosine (5mC) to form 5-hydroxymethylcytosine (5hmC) during demethylation (Gu et al. 2011). Intriguingly, the 5hmC regulated by TET3 substantially overlaps with genes involved in ZGA and is distributed in the promoters, enhancers, and gene bodies of those genes. The diminished 5hmC at specific gene loci, which potentially associate with their restricted development in vitro (Yan et al. 2023). The TET3-deficient signature, in conjunction with the signature arising from activating mutations in DNMT1, which normally opposes TET3, is characterized by hypermethylation and unexpected associations with differentially methylated regions that may be associated with disease pathogenesis (Levy et al. 2021). Epigenetic remodeling is crucial to embryonic development; deficiencies in epigenome remodeling can severely impair embryo development and even lead to lethality (Du et al. 2022). Approximately 40% of the mouse genome is comprised of TEs, with about 10% consisting of ERV sequences (Sakashita et al. 2023). MERVL-derived sequences contribute to preimplantation embryo development and the expression of stage-specific genes during the 2-cell stage. While MERVL expression is commonly used as a marker for totipotency, its role in mouse embryogenesis has remained unclear. Endogenous retroviruses (ERVs) are ancient viral sequences that have been integrated into the genomes of germline cells and passed on from generation to generation. During embryonic development, stages such as the zygote and blastocyst exhibit a large amount of ERV expression. The reasons behind this high expression are not entirely clear, but it correlates with the fact that these stages require significant changes in gene expression, and ERVs could play a role in driving those changes (Wang et al. 2024). Murine endogenous retrovirus-L (MERVL), an ERV, is transiently upregulated at the 2-cell stage around the time of ZGA and drives the expression of many transcripts specific to ZGA and totipotence (Percharde et al. 2018). Is the high overlap between MERVL expression and DNA demethylation timing merely a coincidence, or is there an underlying regulatory mechanism at play?

Currently, there is no relevant reports on how MERVL regulates the epigenetic aspects of embryos and affects their development. ART may affect fetal DNA methylation as the timing of ART procedures coincides with the extensive epigenetic remodeling occurring between fertilization and embryo implantation (Menezo et al. 2019; Håberg et al. 2022; Liu et al. 2021). The development of omics provides insights into the key features of the methylome of early embryos (Guo et al. 2014; Zeng and Chen 2019). MERVL shows a high correlation with the level of DNA methylation (Zhang et al. 2022; Jia et al. 2024) and these reports provide a theoretical basis for our research. In this study, we used our well-constructed model of oxidative damage in IVF mouse embryos to explore epigenetic changes in IVF-derived offspring, particularly the effect of ROS on genome-wide DNA methylation. Given the existing knowledge gaps and the potential implications of oxidative stress on embryogenesis, we hypothesized that oxidative damage in early embryos affects the expression of MERVL, and that abnormal expression of MERVL disrupts DNA methylation reprogramming. Our research provides deeper insights into understanding the mechanisms of embryonic development and the epigenetic landscape.

## Materials and methods

### Animals, IVF, and oxidative damage model

C57BL/6 mice (males: 3–6 months old; females: 6–8 weeks old) were purchased from Beijing Vital River Laboratory Animal Technology Co., Ltd. (Beijing, China) and housed in the Laboratory Animal Center of Shantou University Medical College under standard conditions (12-h light/dark cycle, 21 ± 2 °C, SPF conditions). All animal experiments adhered to the guidelines of the Medical Animal Care & Welfare Committee and were approved by the Laboratory Animal Ethics Committee of Shantou University Medical College. As previously described, sperm were collected from the caudae epididymis of male mice and transferred to capacitation media (Nanjing Aibei Biotechnology Co., Ltd.), incubated for 1 h at 37 °C with 5% CO₂. Female mice were superovulated via sequential injections of 10 IU of pregnant mare serum gonadotropin (PMSG, Ningbo Second Hormone Factory) and 10 IU of human chorionic gonadotropin (HCG, Ningbo Second Hormone Factory), administered 48 h apart. The fertilization dishes and embryo culture dishes were prepared with human tubal fluid medium (SAGE, ART-1020) supplemented with 0.4% bovine serum albumin (Sigma, A1933) to form 35 μl microdrops, which were then covered with sterile paraffin oil (Sigma, M5310). These were prepared in advance and equilibrated in an incubator. Each microdrop containing an oocyte was supplemented with capacitated sperm and incubated for 6 h at 37 °C with 5% CO₂, 35 μl per drop and 10 embryos were cultured in it. Two-cell embryos were collected at 24 h post-insemination (hpi), and blastocysts were collected at 96–108 hpi.

The oxidative stress-damaged mouse zygote model was established as described in our previous study. Zygotes were treated with embryo culture medium containing 0.03 mM H_2_O_2_ for 30 min at 37 °C at 7 hpi, and subsequently cultured in fresh embryo culture media for the remainder of the experiment. To explore the impact of DNA methylation on MERVL expression, the embryos after fertilization were cultured in a medium containing 50 nM 5-Azacytidine (Sigma-Aldrich A3656) (Giaccari et al. 2024).

### Electroporation interferes with MERVL expression

Antisense oligonucleotides (ASO), single-stranded oligonucleotide molecules composed of 15–25 nucleotides, bind to complementary target mRNA by base complementary pairing to inhibit target gene expression. In this study, the phosphorothioate-modified antisense oligonucleotide (S-oligo) of the MERVL sequence was designed to be complementary to 20 bases and hybridize with nucleotides 525 to 544 of the MERVL mRNA (GenBank accession number Y12713) which contains the start codon. The sequence of the antisense MERVL S-oligo is 5ʹ-GATTCATCCTTGTACTTCTG-3ʹ (GenePharma). Then the ASO-targeted sequences were subjected to mutation through inverse PCR in the presence of PrimeSTAR GXL DNA polymerase and specific primer sets (GenePharma). Electroporation was carried out using the Neon™ Transfection System. Briefly, 10 μl of the corresponding kit (MPK1025) was utilized. Solution A was used to prepare 20 μM ASO. Each time, 200 zygotes were transfected with an electrical voltage of 30 V, 5 ms pulse length, and 100 ms pulse interval. For the rescue experiment, the ASO-resistant RNA was co-transfected into the zygotes with 20 μM ASO (mixed) at a final concentration of 200 ng/μl.

### TdT-mediated dUTP nick-end labeling (TUNEL) assay

To analyze blastocyst apoptosis, a TUNEL assay was carried out in line with the manufacturer's instructions by using the RiboAPO™ One-Step TUNEL apoptosis detection kit (red) (RiboAPO, C11026). Zona pellucidae were removed using Tyrode’s acid solution (Sigma, T1788), and embryos were fixed in 4% paraformaldehyde and permeabilized with 0.5% Triton X-100 for 30 min. Embryos were treated with fluorescein-conjugated dUTP and terminal deoxynucleotidyl transferase for 1 h in the dark. The reaction was terminated by washing with TPBS for 15 min, followed by staining with DAPI. ImageJ (NIH Image, Bethesda, MD) was used to quantify the fluorescence intensity of TUNEL-positive cells. The data were derived from three independent experiments, with five blastocysts in each group. To ensure comparability, identical conditions, including the same confocal microscope settings, were used for each group.

### Digital mRNA with pertUrbation of genes (EpiTM DRUG-seq)

EpiTM DRUG-seq is a method optimized for low-input cells and tissues, efficiently capturing mRNA poly(A) tails using oligo dT beads with barcode labels and Unique Molecular Identifiers (UMI) sequences for reverse transcription and library construction, without RNA extraction(Ye et al. 2018).

Mouse 2-cell embryos were collected with three replicates per group, comprising 200 2-cell embryos per sample. cDNA was preamplified for all groups using the SMART-Seq2 protocol (Guangzhou Epibiotek Co., Ltd). Data filtering and quality control were performed as per the protocol. Bowtie2 software was used to align the filtered clean reads with the reference genomes, yielding uniquely mapped reads for further analysis. Gene and TE expression were assessed according to genomic annotation. FPKM (Fragments Per Kilobase Million Reads) was calculated for normalization, and differentially expressed genes (DEGs) were identified through bioinformatics analysis.

### Single-cell Whole-genome bisulfite sequencing (scWGBS)

Single-cell genome-wide bisulfite sequencing is a detection method for genome-wide methylation of extremely small amounts of cells, which helps to uncover the epigenetic mechanisms of early embryonic development (Geek gene, Beijing). For the detection, 50 2-cell embryos per sample were utilized, with four replicates set for each group. Bisulfite conversion was performed in a 150 ml reaction (125 μl CT conversion reagent, 20 μl nuclease-free water, and 5 μl single-cell lysate) using the EZ-96 DNA Methylation-Direct™ MagPrep kit (Invitrogen). Purified DNA underwent two rounds of amplification, incorporating 15 μM Universal PCR Primer (NEB), 15 μM Index Primer (NEB), and 2 × Kapa Hifi Mix (Smallwood et al. 2014). The quality-assured libraries were utilized for paired-end deep sequencing on an Illumina HiSeqX-ten sequencer. All clusters passing the filter were converted into FASTQ files using the standard Illumina pipeline. Modified C bases were distinguished, and high-throughput sequencing technology was employed to compare the methylation status of CpG/CHG/CHH sites against the reference sequence.

### Western blot

Two-cell embryos were extracted in batches. For each batch, 200–300 cells were collected from each group and immediately cryopreserved in a − 70 °C freezer. RIPA lysis solution containing protease and phosphatase inhibitors was used to prepare lysates from 1000 two-cell embryos (control and H₂O₂-treated groups). Protein samples were separated via SDS-PAGE, transferred to PVDF membranes, and probed with primary antibodies overnight at 4 °C. Secondary antibodies conjugated to HRP were applied for an additional hour at room temperature. A chemiluminescence kit was used to visualize protein bands, and BandScan 5.0 software (Glyko, Novato, CA, USA) was used to calculate the optical densities of the bands.

### Immunofluorescence analysis (IF)

IF staining protocol was previously described by our research group. Embryos were digested with Tyrode’s solution to remove the zona pellucida, fixed in 4% paraformaldehyde for 30 min, and permeabilized with 0.5% Triton X-100 in phosphate-buffered saline for 30 min. After blocking with sealing fluid for 1 h, primary antibodies were applied overnight at 4 °C. Following this, secondary antibodies were applied for 1 h at room temperature. After incubation with the second antibody, embryos were stained with DAPI for 15 min, washed with PBST. Fluorescence staining was immediately detected under a confocal laser scanning microscope (Leica, SpectraPlex8). ImageJ (NIH Image, Bethesda, MD) was used to quantify the fluorescence intensity. The data were obtained from three independent experiments, with five embryos in each group. To ensure comparability, identical conditions, including the same confocal microscope settings, were used for each group.

The 5mC and 5hmC double staining protocols differed slightly from conventional immunofluorescence. An additional incubation step in 3 M HCl solution for 30 min and subsequent neutralization in Tris–HCl (pH 8.0) for 10 min was included, while all other steps followed standard IF procedures. Subsequently, the female and male pronuclei can be distinguished based on their size and position. Generally, the male pronucleus is slightly larger than the female pronucleus and is positioned closer to the second polar body.

The primary antibodies used included 5mC (1:200; Epigentek), 5hmC (1:200; Active Motif), MuERV-L Gag (1:200; Beyotime), OCT4 (1:200; Abcam), anti-γH2AX (1:100; Santa Cruz), anti-DNMT1 (1:200; CST), and anti-TET3 (1:200; CST). Secondary antibodies included goat anti-rabbit, Conjugation: FITC (1:200; Abcam, ab6717) and goat anti-mouse, Conjugation: Alexa Fluor 594 (1:200; Abcam, ab150116).

### Quantitative real-time polymerase chain reaction (RT-PCR)

A total of 150 two-cell embryos were prepared, and total RNA was extracted using the RNAprep Pure Micro Kit. cDNA was synthesized from RNA samples using FastKing gDNA Dispelling RT SuperMix. Real-time PCR was performed using Takara RR420Q TB Green® Premix Ex Taq™ (RR420Q, Takara, Japan) on a RT**-**PCR Detection System (CFX96, Bio-Rad, USA), with GAPDH as a control for each sample. Relative mRNA expression levels of target genes were calculated using the 2 − ΔΔCt method. Primers are detailed in Supplementary Table 1.

## Results

### Oxidative stress induce development arrest, DNA damage, apoptosis in IVF mouse embryos

**Fig. 1:**
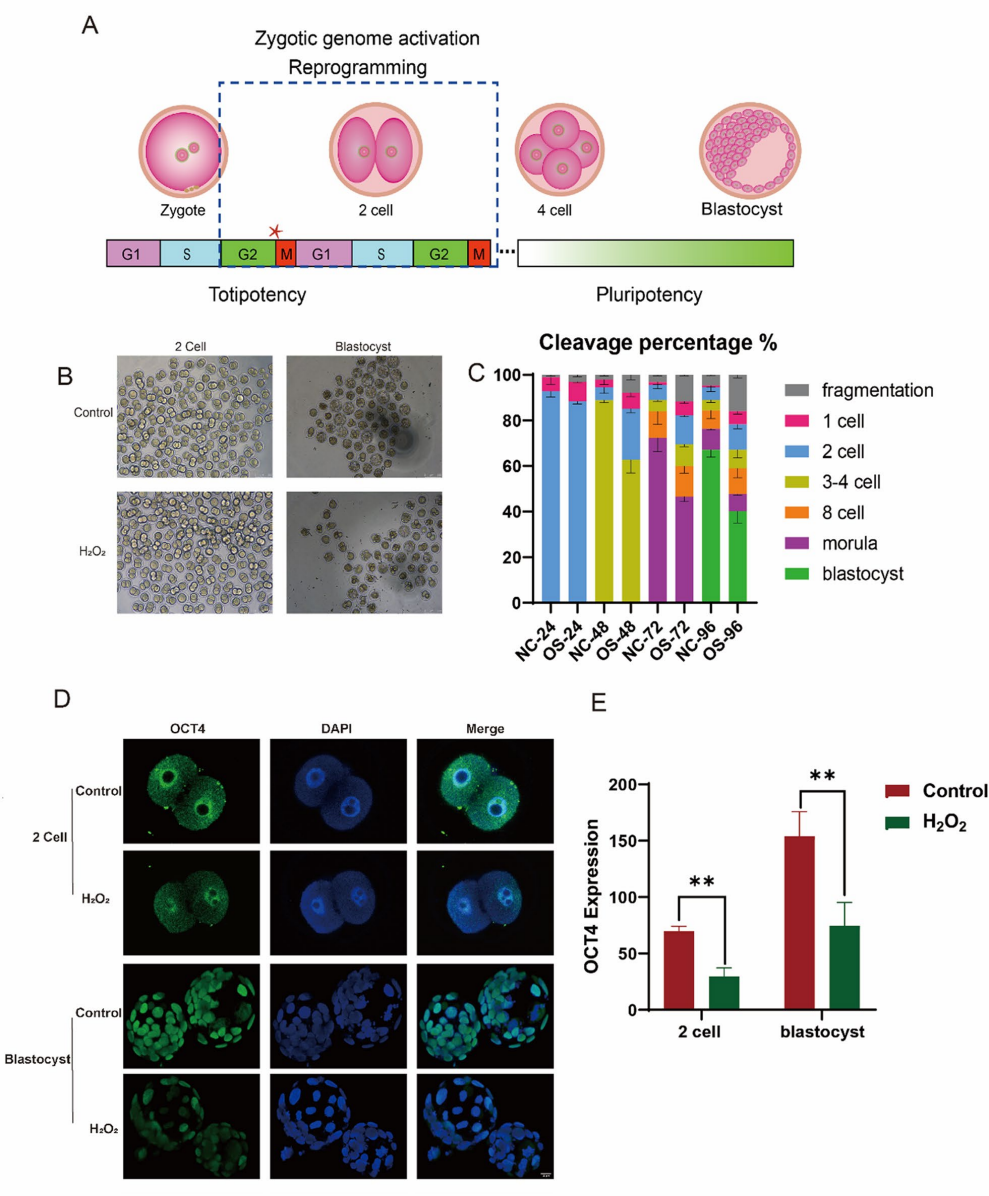
Impact of oxidative damage on embryonic development. **A** Oxidative stress in fertilized eggs causes G2/M phase arrest during the first cleavage, occurring in the ZGA phase, when the entire genome undergoes reprogramming. **B** Cleavage of 2-cell and blastocyst stages in IVF embryos after exposure to oxidative stress. **C** Effects of oxidative stress on the cleavage frequency of embryos in IVF mice. NC: the Control group; OS: the H_2_O_2_ group. **D** Expression levels of OCT4 protein in 2-cell and blastocyst stages following oxidative damage to fertilized embryos. **E** Representative immunofluorescence images showing OCT4 expression in 2-cell and blastocyst stages. Data are presented as means ± SDs, collected from three independent experiments, n = 5. Differences between groups were analyzed using Student’s t-test. *P < 0.05 vs. control; **P < 0.01; ***P < 0.001. G2/M: gap 2/mitosis; ZGA: zygotic gene activation; IVF: in vitro fertilization; OCT4: octamer-binding transcription factor 4

Embryo development initiated with the fusion of sperm and egg (oocyte) to form a fertilized egg, which then cleaved into a 2 cell stage. Most zygotes required in vitro culture until they developed into blastocysts before transfer. During this phase, ZGA occurred, enabling the embryo to attain totipotency and the ability to differentiate into various cell types, including extra-embryonic tissues and a complete organism (Fig. 1A). Early embryonic development involved whole-genome reprogramming, making embryos highly sensitive to external environmental factors. Our previous findings confirmed a G2/M block in fertilized eggs subjected to oxidative stress, leading us to speculate that oxidative damage affected early embryonic ZGA and subsequently embryonic development (He et al. 2020). We observed a decrease in the cleavage of 2-cell embryos after exposure to oxidative stress, although this change was not statistically significant. There was a 15% reduction in blastocyst development, indicating that oxidative damage might lead to more cells remaining in the 2-cell stage without progressing (Fig. 1B, C). Furthermore, oxidative stress resulted in decreased OCT4 protein expression, which may alter gene expression patterns and ultimately influence cell fate (Fig. 1D, E).

**Fig. 2:**
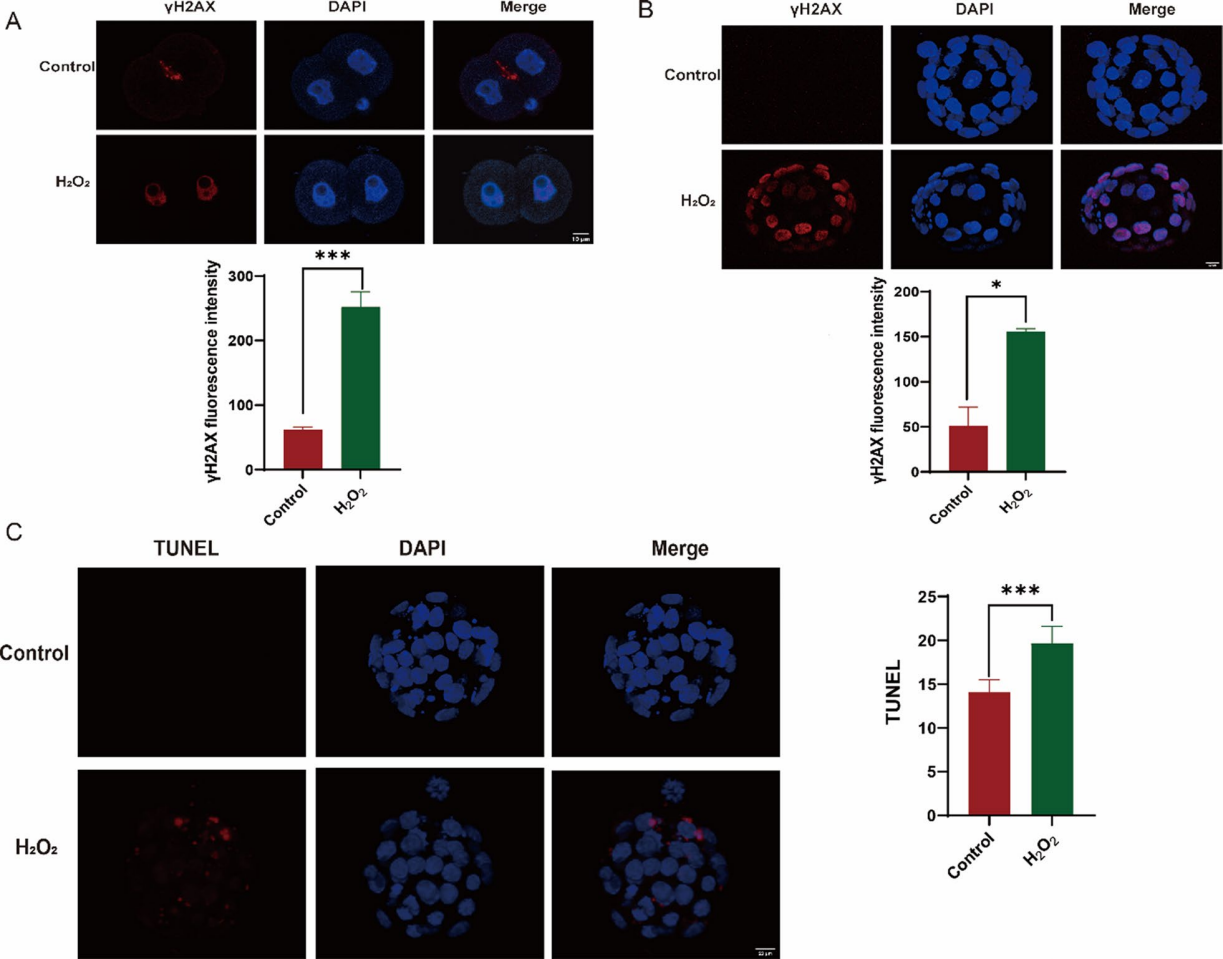
Oxidative damage induces increased apoptosis. **A** Oxidative damage enhances γH2AX expression during the 2-cell stage. **B** Oxidative damage increases γH2AX expression in the blastocyst stage. **C** TUNEL assay indicating apoptosis in blastocysts after oxidative stress. Data are presented as means ± standard deviations (SD), collected from three independent experiments, each involving more than five embryos. Differences between groups were analyzed using Student’s t-test. *P < 0.05 vs. control; **P < 0.01; ***P < 0.001. γH2AX: gamma histone H2AX; TUNEL: terminal deoxynucleotidyl transferase dUTP nick end labeling; IVF: in vitro fertilization

Cells activated repair signaling pathways when DNA was damaged, leading to the formation of γH2AX, which accumulated near DNA break sites and recruited repair proteins. If the damage was irreparable, cells might undergo apoptosis to eliminate those with potentially dangerous mutations. DNA damage triggered signaling pathways and caspase activation, leading to cellular changes and apoptosis. γH2AX serves as a marker for DNA damage, reflecting cellular health. We found that oxidative damage led to DNA damage as early as the 2-cell stage (Fig. 2A, B). In the embryos that developed to the blastocyst stage, we compared the fluorescence intensity of TUNEL (Terminal—deoxynucleotidyl Transferase Mediated Nick End Labeling) in the blastocysts of the two groups. Compared with the control group, the apoptosis of cells in the hydrogen peroxide (H₂O₂) treatment group increased (Fig. 2C).

### Possible mechanism of oxidative damage in zygotes leading to apoptosis

**Fig. 3:**
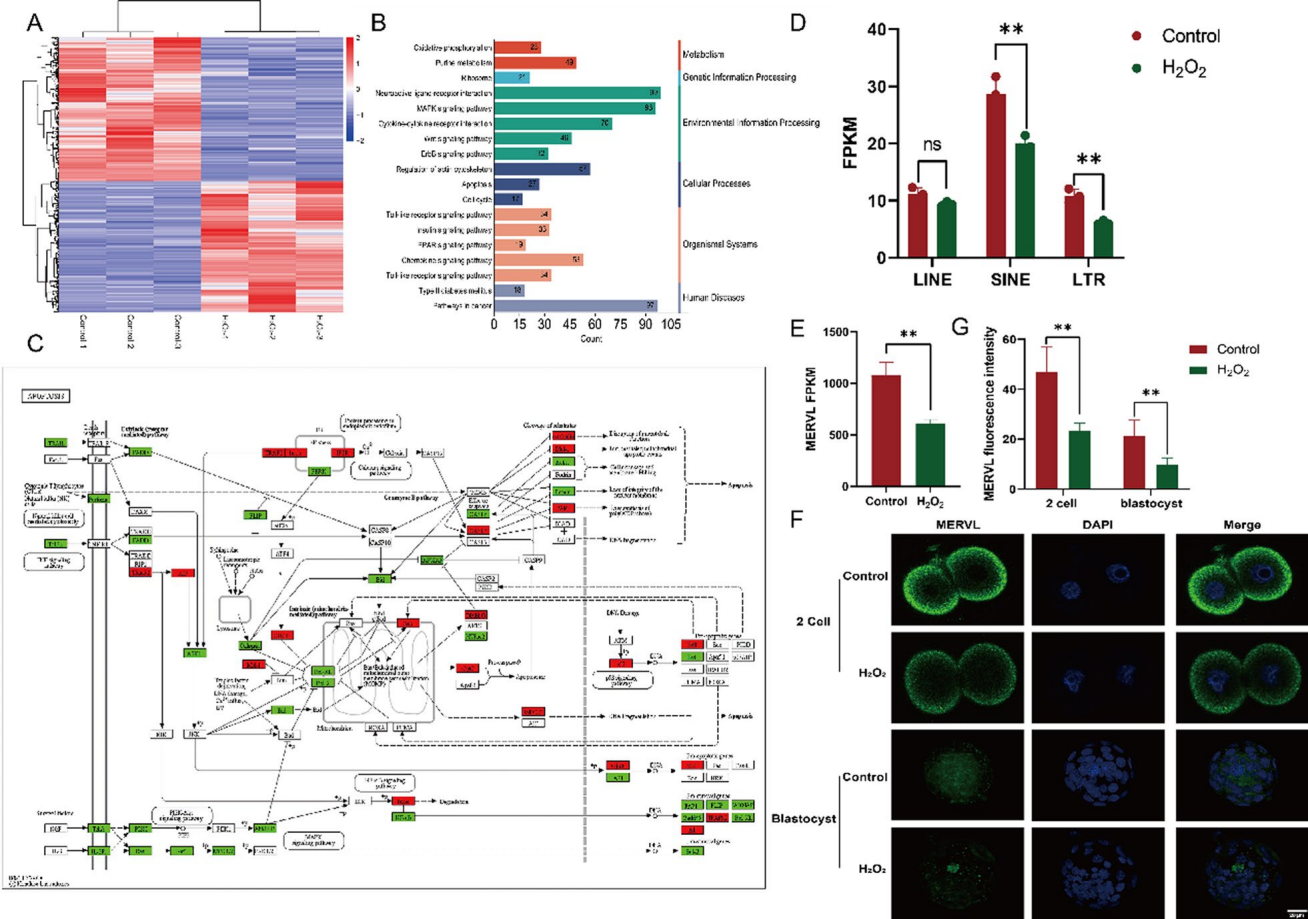
DEGs associated with oxidative damage in zygotes. **A** Volcano plot showing DEGs in 2-cell embryos following oxidative damage to zygotes. **B** Functional analysis of DEGs. **C** DEGs enriched in the apoptotic pathway, with up-regulated genes depicted in red and down-regulated genes in green. **D** The impact of oxidative damage on the expression of TEs. **E** The impact of oxidative damage on MERVL expression in fertilized eggs. **F** Immunofluorescence analysis was used to analyze the impact of oxidative damage on MERVL expression. **G** Statistical results representing the expression of MERVL in different groups after oxidative damage in early embryos. Bar = 20 μm. Data are presented as means ± SDs, collected from three independent experiments, each involving more than five embryos. Differences among groups were analyzed using Student’s t-test. *P < 0.05 vs. control; **P < 0.01; ***P < 0.001. DEGs: differentially expressed genes

Using EpiTM DRUG-seq, we identified DEGs associated with oxidative damage in 2 cell embryos, with 1,229 up-regulated and 1173 down-regulated genes (Fig. 3A). Consistent with previous research, functional analysis of DEGs revealed that oxidative damage primarily affects oxidative phosphorylation, Wnt signaling pathways, apoptosis, and insulin signaling pathways (Fig. 3B). This damage contributes to early embryo apoptosis (Fig. 3C). Embryonic development necessitates precise regulation; any abnormalities at any stage can influence embryonic development and the health of offspring. We analyzed TEs and found that oxidative stress in zygotes decreased the expression of inversion sequences, long terminal repeats (LTR), non-LTR retrotransposons (LINE), and SINE. MERVL, a marker of pluripotency that increased during ZGA, exhibited significantly reduced expression following oxidative damage in fertilized eggs (Fig. 3D–F).

### MERVL retrotransposons were essential for preimplantation embryo development

**Fig. 4:**
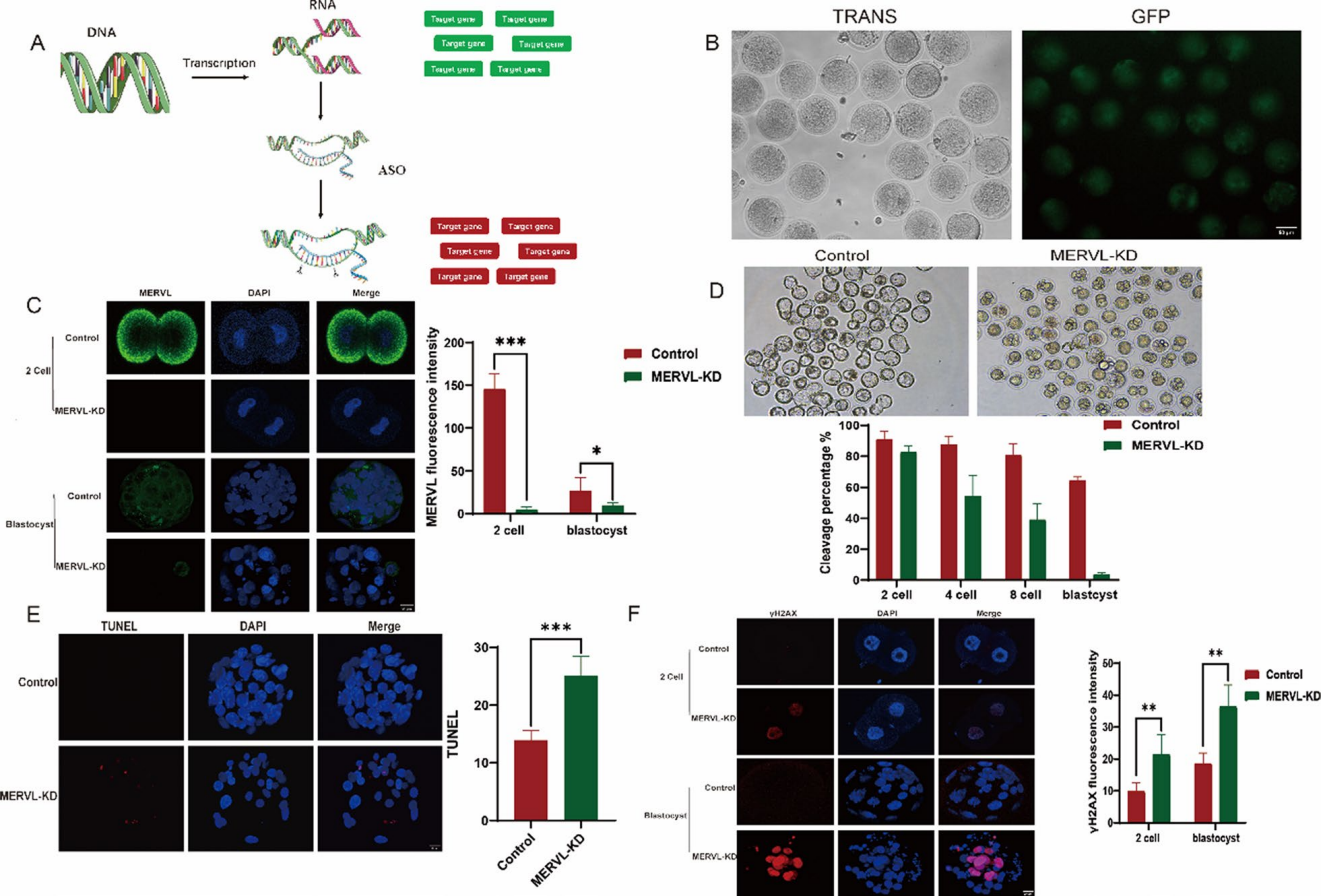
MERVL plays a crucial role in maintaining the genome stability of pre-implantation embryos. **A** Knockdown of MERVL using ASOs. **B** Electroporation of fluorescently labeled controls using a voltage of 800 V, pulse duration of 20 ms, repeated twice. **C** Representative images of immunofluorescence staining for MERVL-Gag protein, counterstained with DAPI, in control and MERVLKD embryos at the late two-cell stage, derived from three independent experiments. Scale bars, 20 µm. **D** Representative phase-contrast images of 4.5 dpc blastocysts in control and MERVL-KD embryos, from three independent experiments. Scale bars, 100 µm. **E** Representative images of immunofluorescence staining for TUNEL apoptosis in control and MERVL-KD embryos at the blastocyst stage, from three independent experiments. Scale bars, 20 µm. **F** γH2AX fluorescence intensity in control and MERVL-KD embryos. Data are presented as means ± SDs. *P < 0.05 vs. control; **P < 0.01; ***P < 0.001. ASO: antisense oligonucleotide; KD: knockdown; γH2AX: gamma H2A histone family member X; dpc: days post-coitum

In this study, we employed antisense oligonucleotides (ASO) to consistently suppress the distributed copies of MERVL and investigate its regulatory mechanisms in embryonic development (Fig. 4A). To enhance transfection efficiency and reduce time, we used the NEON transfection system, which streamlined the electroporation process. This system effectively introduced green fluorescent protein (GFP) mRNA into mouse zygotes (Fig. 4B). By introducing the MERVL plasmid into fertilized eggs using this method, we observed a significant decrease in MERVL expression during the crucial 2-cell and blastocyst stages (Fig. 4C). Next, we examined the impact of MERVL knockdown (MERVL-KD) using ASO on preimplantation development. Notably, the absence of MERVL did not affect the frequency of two-cell division (Fig. 4D). However, it led to a significant decrease in blastocyst formation (Fig. 4D), indicating that MERVL is important for successful blastocyst development. Additionally, we observed an increase in TUNEL apoptosis, indicative of programmed cell death, in MERVL-KD embryos (Fig. 4E). We also explored the expression levels of γH2AX, a marker for DNA damage, in MERVL-KD blastocysts. Surprisingly, these blastocysts exhibited upregulated γH2AX expression (Fig. 4F), providing compelling evidence that the absence of MERVL induces DNA damage and subsequent apoptotic events during the preimplantation stage.

### Oxidative stress leads to abnormal DNA methylation in IVF mouse embryos

**Fig. 5:**
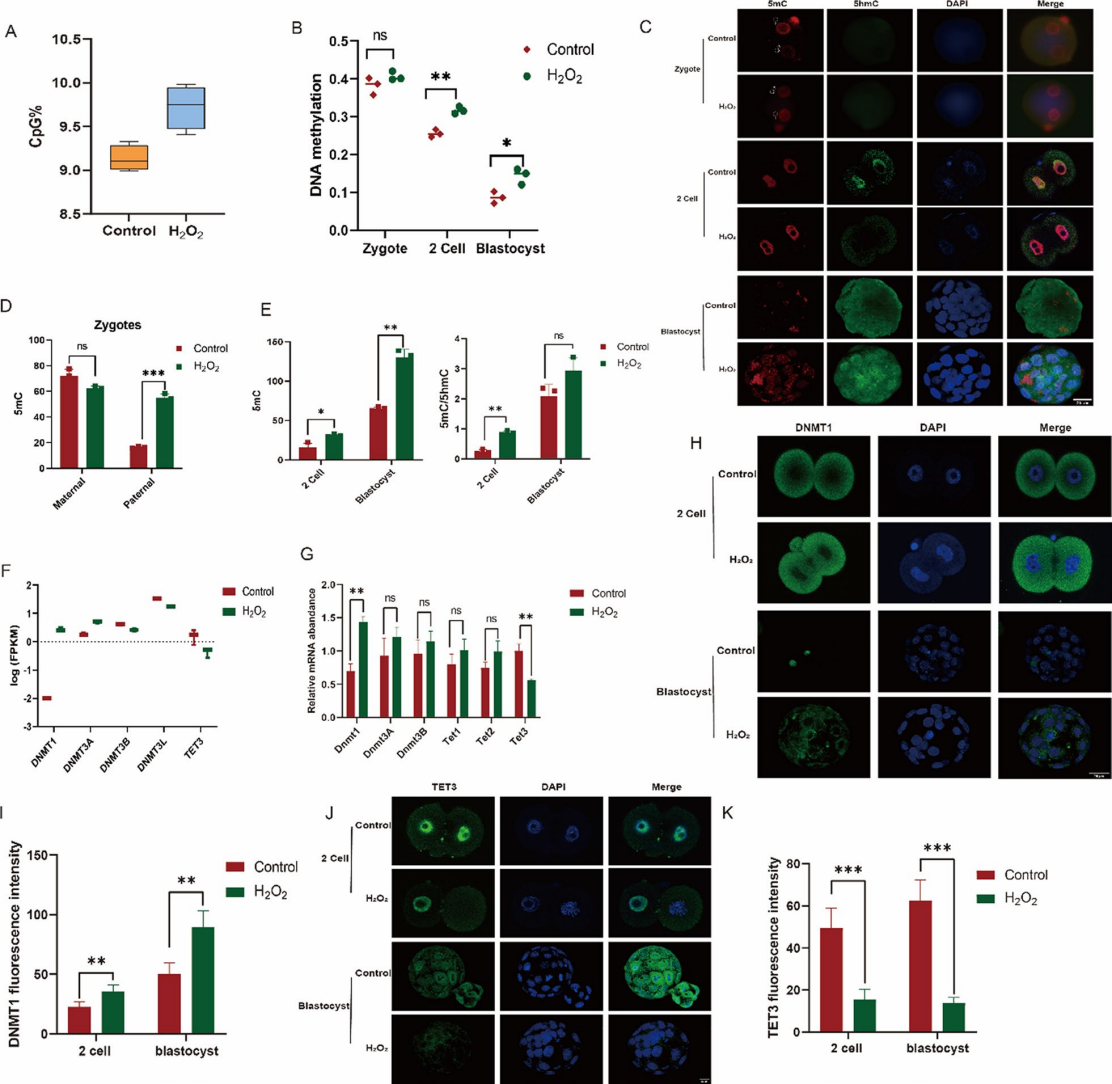
Oxidative damage to zygotes results in alterations of DNA methylation. **A** WGBS showing alterations in DNA methylation following oxidative damage to zygotes. **B** Statistical analysis of 5mC content at various stages of pre-implantation embryos after oxidative damage. **C** Immunofluorescence analysis of DNA methylation following oxidative damage to zygotes. **D** Statistical analysis of immunofluorescence intensity in the female and male pronuclei after oxidative damage. **E** Statistical analysis of immunofluorescence intensity in embryos at the two-cell and blastocyst stages following oxidative damage. **F** Expression analysis of DNA methyltransferases and demethylases after oxidative damage using drug sequencing (Drug-seq). **G** Expression analysis of DNA methyltransferases and demethylases following oxidative damage using RT-PCR. **H** Expression levels of DNMT1 in two-cell and blastocyst embryos after zygote oxidative damage. **I** Statistical analysis of fluorescence intensity for DNMT1 expression. **J** Expression levels of TET3 in two-cell and blastocyst embryos following zygote oxidative damage. **K** Statistical analysis of fluorescence intensity for TET3 expression. WGBS: whole genome bisulfite sequencing; 5mC: 5-methylcytosine; Drug-seq: drug sequencing; DNMT1: DNA methyltransferase 1; TET3: ten-eleven translocation 3

Using WGBS, we examined CpG methylation patterns across the genome in early-stage embryos and observed a significant increase in global methylation levels following oxidative damage (Fig. 5A). Statistical analysis revealed an elevation in 5mC levels at each preimplantation stage following oxidative damage to zygotes (Fig. 5B). To determine when changes in DNA methylation occur post-oxidative damage, we employed IF to detect 5mC and 5hmC levels starting from the fertilized egg (Fig. 5C). Our findings indicated that DNA methylation alterations began during the fertilized egg stage. After fertilization, a global DNA demethylation event was expected; however, the male pronucleus in zygotes failed to undergo timely demethylation following oxidative damage, resulting in higher 5mC levels compared to the control group (Fig. 5D). This discrepancy persisted through subsequent cleavage divisions and remained elevated in preimplantation embryos (Fig. 5E). Analyzing enzymes involved in regulating DNA methylation, we discovered that oxidative damage led to increased expression of DNMT1, promoting DNA methylation, while the expression of TET3, which promoted DNA demethylation, decreased (Fig. 5F, G). Proteins play a crucial role in biological functions; therefore, we analyzed differential protein expression influencing DNA methylation. Following oxidative damage at the 2-cell and blastocyst stages, we observed increased DNMT1 expression (Fig. 5H, I) and decreased TET3 expression (Fig. 5J, K).

### MERVL regulated DNA methylation changes in early embryos with oxidative damage through TET3

**Fig. 6:**
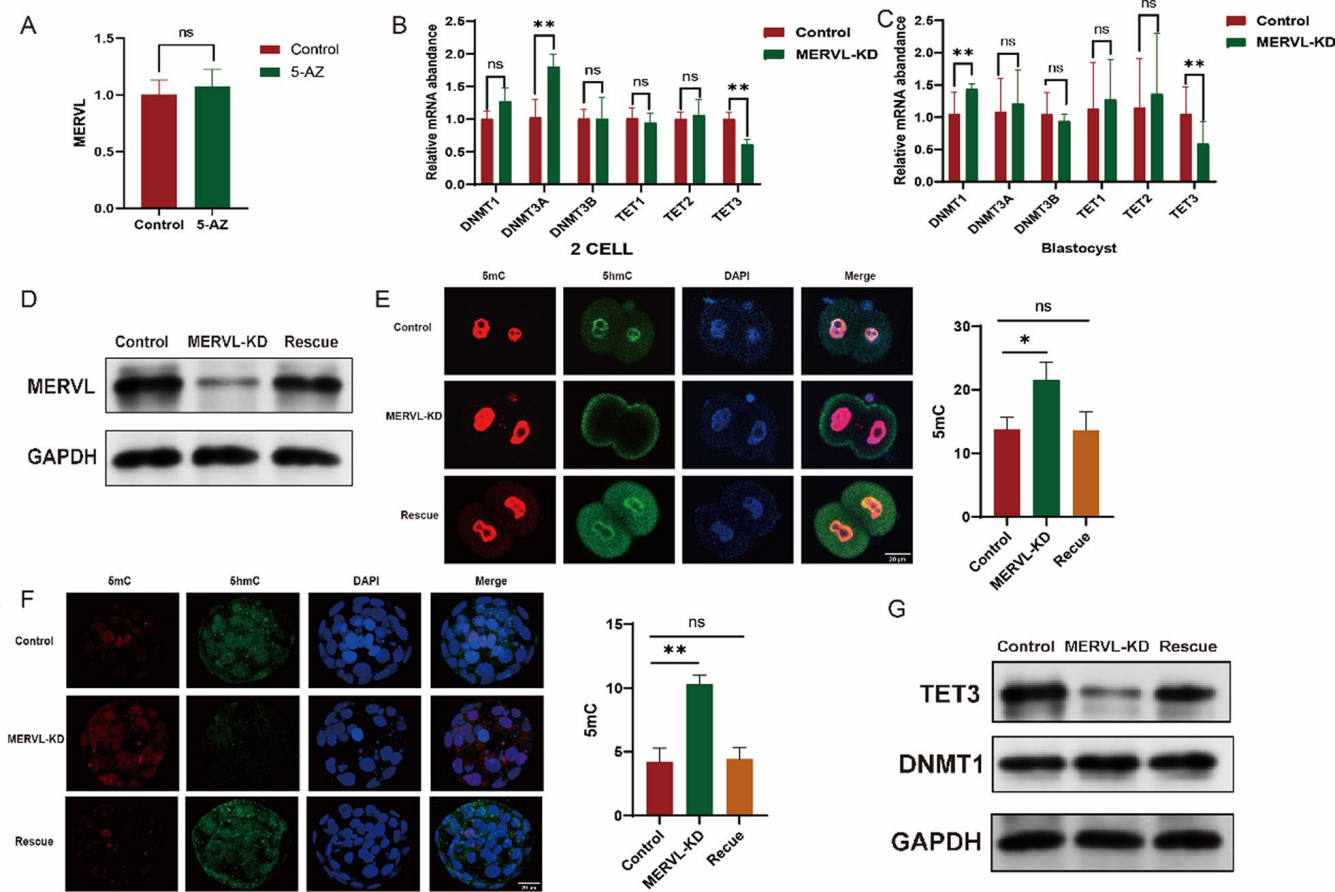
MERVL regulates DNA methylation changes in early embryos. **A** The expression of MERVL is unaffected by DNA methylation levels. **B** Expression of DNA methylation enzymes in two-cell embryos following MERVL-KD. **C** Expression of DNA methylation enzymes in blastocysts after MERVL-KD. **D** Establishment of the MERVL-KD and rescue groups. **E** DNA methylation levels in two-cell embryos from the MERVL-KD and rescue groups. **F** DNA methylation levels in blastocysts from the MERVL-KD and rescue groups. **G** Expression levels of DNMT1, TET3 in the MERVL-KD and rescue groups. MERVL: murine endogenous retrovirus-like; MERVL-KD: MERVL knockdown; DNMT1: DNA methyltransferase 1; TET3: ten-eleven translocation 3

Is MERVL regulated by DNA methylation? We hypothesized whether the activity of the endogenous retrovirus-like element MERVL is linked to global genome demethylation processes. We added 5-Azacytidine to the culture medium to inhibit DNA methylation; however, we did not observe the expected differences in MERVL expression (Fig. 6A). Nevertheless, we noticed a high temporal overlap between MERVL and DNA methylation changes, prompting us to further hypothesize that MERVL influences DNA methylation dynamics. Interestingly, upon knockdown MERVL, we observed changes in the expression of DNA methyltransferases. During the 2-cell and blastocyst stages, TET3 expression decreased, while DNMT3A expression increased during the 2-cell stage, with no significant change in DNMT1 expression. However, in the blastocyst stage, DNMT1 expression increased, while DNMT3A expression remained unchanged (Fig. 6B, C). We conducted rescue experiments following MERVL knockdown to assess the impact on early embryo DNA methylation (Fig. 6D). We found that knocking down MERVL prevented the conversion of 5mC to 5hmC in the 2-cell stage, while rescue experiments induced this conversion (Fig. 6E). In the blastocyst stage, we observed a similar phenomenon: the MERVL knockdown group exhibited reduced 5hmC levels, while rescue experiments increased 5hmC levels (Fig. 6F). Thus, we propose that MERVL influences DNA methylation through TET3. After disrupting MERVL, we noted a reduction in TET3 expression. Subsequently, rescue experiments resulted in increased TET3 expression, suggesting that MERVL regulates TET3 expression (Fig. 6G).

## Discussion

ART treatments are linked to various adverse obstetric and perinatal outcomes, and they increase long-term health risks for offspring (Cui et al. 2020; Elhakeem et al. 2022; Li et al. 2024). ART procedures can induce epigenetic and genetic changes in gametes and early embryos, contributing to these adverse effects (Lu et al. 2024; Liu et al. 2024).

In this study, we elucidated partial mechanisms of cell fate and epigenetic reprogramming in the context of oxidative damage to zygotes. During the early division phase (e.g., the 2-cell phase), demethylation can occur, followed by re-methylation during the blastocyst phase. Environmental factors can significantly impact embryo development by altering DNA methylation patterns (Mitchell et al. 2016; Toraño et al. 2016; Canovas et al. 2017). In ART procedures, oxidative stress negatively affects gametogenesis, fertilization, and fetal development, potentially leading to lasting consequences for offspring (Yang et al. 2020; Shah et al. 2024; Chen and Riggs 2011). ERVs, comprising about 8% of the human genome, can be neutral, harmful, or even beneficial (Gale Hammell and Rowe 2020). Using a well-established model of oxidatively damaged IVF mouse embryos, we found that transposon element expression, particularly MERVL, is abnormal after oxidative damage to fertilized eggs. Our analysis revealed that knocking down MERVL decreased the blastocyst formation frequency and increased apoptosis. MERVL appears to induce apoptosis and facilitate the nuclear entry of DNMT1 to repair damaged DNA. Additionally, we discovered that MERVL influences DNA demethylation through TET3.

Oxidative damage reduces MERVL expression in embryos. The MERVL retrotransposon is transiently activated in 2-cell mouse embryos and is a widely recognized indicator of ZGA and totipotency (Xie et al. 2022; Ishiuchi et al. 2015; Choi et al. 2017; Yang et al. 2024). These retrotransposons likely function as cis-regulatory elements, aiding in gametogenesis and early development (Flemr et al. 2013; Modzelewski et al. 2021). Chromatin maintenance and remodeling are crucial processes that operate alongside DNA repair, replication, or transcription to ensure cellular survival and flexibility (Torres-Arciga et al. 2022). Excess ROS can lead to mitochondrial dysfunction and DNA damage. Research shows that mitochondrial division inhibitor-1 (Mdivi-1) affects pre-implantation mitochondrial function, resulting in increased ROS generation and subsequent epigenetic abnormalities in embryos, as well as decreased stability of ZGA markers such as ZSCAN4 and MERVL (Li et al. 2021). Our previous work demonstrated that high lipid-mediated oxidative damage affects MERVL expression through epigenetic inheritance (Huang et al. 2022). Interestingly, while high ROS levels can induce a transition of ESCs into a 2-cell-like state with enriched 2C-specific ZGA transcripts (Zhang et al. 2019), different results may stem from varying degrees of oxidative stress. For instance, high osmotic environments can generate excessive ROS, activating the ATR checkpoint and promoting 2-cell-like cell induction, with elevated expression levels of pluripotency-related genes such as Dux, MERVL, and Zscan4 observed after recovery from high osmotic stress for 6 h (Canat et al. 2023). Our findings indicated that MERVL knockdown affected blastocyst formation, increased γH2AX expression (a DNA damage marker), and raised TUNEL apoptosis levels. Furthermore, we observed no early changes in DNMT1 expression post-MERVL knockdown, but increased DNMT1 expression during the blastocyst stage suggests that MERVL knockdown may recruit DNMT1 for DNA repair.

Both animal and human studies indicate that ART is associated with changes in DNA methylation within embryonic and extraembryonic tissues (Peral-Sanchez et al. 2021). S-adenosylmethionine (SAM) serves as a major methyl donor in methylation reactions (Laurino and Tawfik 2017). When the balance between antioxidant and oxidative systems is disrupted, excessive ROS can alter the availability of cofactors such as SAM and O_2_, which drive the epigenetic control of gene expression (Hitchler and Domann 2007; Hussain et al. 2021). The quiescent ESCs exhibit a notable decrease in SAM, demonstrating molecular characteristics akin to 2C embryos, with increased expression of MERVL, 2C-specific genes, and trophoblast master regulators (Guo et al. 2014; Khoa et al. 2024). DNA methylation is a critical epigenetic modification involved in suppressing TEs and ERVs. However, the transient expression of MERVL in 2-cell embryos appears to be independent of the levels of DNA methylation (Schoorlemmer et al. 2014). Is there any relationship between MERVL and DNA methylation? DNA methylation has been commonly acknowledged as a crucial epigenetic modification involved in the suppression of TE and ERV. However, the temporary expression of MERVL in two-cell embryos appears independent of DNA methylation levels (Fu et al. 2019; Eckersley-Maslin et al. 2016). Conversely, other studies suggest that ESCs cycle through transient hypomethylated states marked by MERVL expression (Eckersley-Maslin et al. 2016; Dahlet et al. 2020). TET3 mediates overall paternal DNA demethylation and subsequent maternal DNA demethylation through replication (Shen et al. 2014). Absence of TET3 leads to significant impairments in oocyte development, maturation, fertilization, as well as in the subsequent cleavage and blastocyst formation proportions (Cheng et al. 2019). In ART contexts, TET3 abnormalities disrupt epigenetic reprogramming in early embryos (Canovas et al. 2017; Montgomery et al. 2024). Our research revealed that early oxidative damage to zygotes affected MERVL transposon expression and global demethylation in embryos by regulating TET3.

Our findings indicated that after oxidative damage to zygotes, MERVL expression decreased. The absence of MERVL led to DNA damage and increased apoptosis, while it also affected the dynamics of DNA methyltransferases, particularly the demethylase TET3. Supplementation of MERVL restored TET3 to normal levels, suggesting that MERVL might drive early embryos to undergo genome-wide demethylation.

## Conclusions

ERVs contribute to ∼10 percent of the mouse genome. While these elements are typically silenced, they exhibit differential expression during various stages of embryonic development. Although MERVL expression is widely used as a marker of totipotency, the role in embryonic development is unclear. Our study showed that oxidative damage in fertilized eggs decreased MERVL expression, and analyzed the effects of MERVL knockdown on embryonic development. Our study provides a basis for the mechanism of oxidative damage in assisted reproductive technology and the conditions of embryonic development.

## Supplementary Information

Additional file 1.

## Data Availability

All data can be obtained by contacting the corresponding author.
